# The Three Kinds of Cod-Liver Oil; Comparatively Considered, with Reference to Their Chemical and Therapeutic Properties

**Published:** 1850-01

**Authors:** 


Art. VI.—*The Three Kinds of Cod-Liver Oil; comparatively considered, with reference to their chemical and therapeutic properties.* By L. J. de Jongh, M. D., of the Hague. Translated from the German, with appendix and cases, by Edward Carey, M. D. To which is added an article on the subject from "Dunglison on New Remedies." Philadelphia: Lea & Blanchard.

Dr. Jongh has investigated the chemical and therapeutic properties of the different kinds of cod-liver oil with a great deal of patience, skill and judgment; much more, indeed, than are usually brought to bear on such subjects. We wish that we could feel the same degree of confidence in Dr. Jongh's testimony to the therapeutic properties of the three kinds of cod-liver oil, that we do in his chemical evidence. The researches of the laboratory may be more accurate, unvarying and satisfactory than the reports of the success of a popular medicine in various forms of disease. But while we must hold many of the statements of Dr. Jongh on the therapeutic value of cod-liver oil, *sub judice*, we feel it incumbent on us to let our readers know what he says. We shall not, however, attempt to carry them a wearisome journey through the chemical paths of the subject, but shall confine our notice to the diseases said to have been cured by the cod-liver oil. These are the details most likely to interest our readers.

Cod-liver oil was known, as a medicine, to Dr. Percival as early as 1771. It was then used for chronic rheumatism. From that time, down to 1822, occasional notices of it occur, but Schenk, who published his report of sixteen cases of rheumatism, cured by this remedy, enjoys the honor of having brought the subject fairly before medical men. In 1826, Schenk reported twenty other cases, which excited much attention. From his observation and experience, he believed the cod-liver oil as much a specific in rheumatism, as quinine is in ague, or mercury in syphilis. From the time of Schenk's publications, down to the present time, hosts of medical men have borne strong testimony to the remedial powers of cod-liver oil. The best effects, after the failure of all other remedies in many cases, are reported in the following forms of disease: rickets, scrofulous caries, fungus articularis in a scrofulous diathesis, rheumatic palsy, pædarthrocace, scrofulous abscess, with extreme emaciation, exostosis with a scrofulous taint, sciatica, and coxalgia. In 1833, Hankel discovered its virtues in tuberculous phthisis, and a great deal of proof has accumulated since, in favor of the employment of the remedy in this disease. Cardialgia and hemicrania of a rheumatic character, we suppose, were relieved by the cod-liver oil, after they had resisted all other remedies. In herpes, and scrofulous eruptions generally, this medicine was found efficacious. Smeet says that a combination of cod-liver oil and hydriodate of potash, renders all other medicine superfluous in confirmed tubercular consumption.

A great deal of evidence is brought forward to show the vast superiority of cod-liver oil over all other remedies in rheumatic and scrofulous disease. In scrofulous ulcers, used externally, this oil is said to have shown great restorative power. In osteo malacia, carious scrofulosa, and caries of the femur, its powers were very satisfactorily witnessed by many physicians. In tumor albus, an affection involving the ligaments, cartilages, and bones of joints, the reports are very favorable to the use of the cod-liver oil. It is also supposed to exert a beneficial influence over leucorrhea and amenorrhea.

The use of this medicine should be discontinued if indigestion, hemorrhage, or diarrhea should come on during the administration of it.

From an examination of a number of tables carefully prepared, and remarkably well reported, in order to settle the question as to which is the best oil for remedial purposes, it is evident that the brown cod-liver oil cured in half the time required under the use of the other two kinds.

The doses of cod-liver oil for children should be from one to two teaspoonfuls two or three times a day. Adults may begin on tablespoonful doses three times a day, and these may be increased to eight or ten tablespoonfuls a day.

It is difficult to disguise the taste. Peppermint produces eructations which renew the taste of the oil. The medicine should be taken after a full meal, and a little

Madeira wine, taken immediately after the oil, assists materially in making it palatable. Various formulas are reported for giving this oil, but each physician may easily make a preparation for himself. We shall not, therefore, encumber our pages with a republication of any of the formulas given.

We have no doubt that in many of the forms of disease of the bone, which we discussed in the December number of this Journal, and the consideration of which is renewed in this number, in an analysis of Mr. Stanley's work, the cod-liver oil will prove a valuable remedy. In a portion of Mr. Stanley's work, held over for future analysis, that gentleman bears strong testimony to the virtues of cod-liver oil.

We thank Dr. Carey for having introduced the labors of Dr. Jongh to the acquaintance of American physicians, and we indulge the hope that these labors will prove to be valuable.

